# The panorama of animal leptospirosis in Rio de Janeiro, Brazil, regarding the seroepidemiology of the infection in tropical regions

**DOI:** 10.1186/1746-6148-9-237

**Published:** 2013-12-01

**Authors:** Gabriel Martins, Walter Lilenbaum

**Affiliations:** 1 Department of Microbiology and Parasitology, Laboratory of Veterinary Bacteriology, Universidade Federal Fluminense, Rio de Janeiro, Brazil

**Keywords:** Animal leptospirosis, Tropical scenario, Infection, Brazil

## Abstract

**Background:**

Leptospirosis is an important disease caused by various serovars of *Leptospira* sp. It can affect humans as well as domestic and wild animals; therefore, it has importance for public health, animal production, and wild species. The aim of this paper is to discuss the epidemiology of animal leptospirosis in Rio de Janeiro, Brazil, as a possible model for other tropical regions. In several studies conducted in the last 20 years, a total of 47 rats, 120 dogs, 875 cows, 695 horses, 1,343 goats, 308 sheep and 351 pigs from all regions of the state, in addition to 107 wild mammals and 73 golden-lion tamarins were tested (MAT) for anti-*Leptospira* antibodies.

**Results:**

Seroreactivity was frequent in all studied species, confirming that the infection is endemic in Rio de Janeiro. Serogroups Icterohaemorrhagiae and Sejroe were the most prevalent in urban and rural scenarios, respectively. This paper reviews the current knowledge on animal leptospirosis in Rio de Janeiro and describes important differences between urban versus rural cycles of the infection in various species.

**Conclusion:**

Identification of the prevailing serogroups and their reservoirs is essential for understanding agent-host-environment interactions under tropical conditions.

## Background

Leptospirosis is an important disease caused by various serovars of *Leptospira* sp. It can affect humans as well as wild and domestic animals; therefore, it has importance for both public health and animal production [1,2].

Several syndromes have been identified in animal leptospirosis. Some species, e.g. dogs and less often horses, tend to present the classic acute disease, which includes the icteric-hemorrhagic syndrome, with fever, pulmonary involvement, and renal failure. Conversely, other species, mainly ruminants, but also swine, usually present the reproductive form of the disease, characterized by abortions and premature or weak offspring, stillbirths and fetal mummification, leading to substantial losses worldwide [3-6].

This infection has been classified into two major groups. The first is determined by strains adapted to and carried by the affected host, which are less dependent on the region or environmental conditions, as topography or rainfall (e.g. serovar Hardjo in cattle or Canicola in dogs), and usually leads to subclinical infection, becoming an important source of infection for humans or other animals. The other group consists of incidental infections caused by strains carried by other domestic and free-living animals, and are more dependent on environmental factors and management practices, which results in direct or indirect contact of the animal with the urine of the reservoirs of the bacterium. That last group constantly leads to an acute and severe leptospirosis syndrome, e.g. serovar Pomona in cattle or Icterohaemorrhagiae in humans or dogs [7,8]. It has been suggested that this second group could be relatively more important in tropical countries than in other regions [1], particularly under conditions of bad hygiene [9]. Though distinct variations in maintenance hosts and the serogroups they carry can occur throughout the world, a basic knowledge of serogroups and their maintenance hosts is required to understand the epidemiology of leptospirosis, either human or animal, in a particular region [6].

Human leptospirosis is usually due to serovars that are maintained by the animal populations of a region, which spread the bacterium on the environment [2,10]. Recent studies conducted in many tropical countries reinforce the complex epidemiological relationship between human/animal leptospirosis. Human beings are not associated as maintenance hosts of any leptospiral serovar; therefore, they consistently present incidental infection [1,6,11,12].

Tropical regions have many particularities that affect the occurrence of the infection, as well as its routes and disease severity [7,13,14]. In addition to geographical conditions and aspects as climate or topography, management factors and husbandry practices, as well as frequency of veterinary assistance, may affect overall seroprevalence and also the serovar distribution [3,9,15,16].

The aim of this paper is to discuss in a broader perspective various results obtained during 20 years in several studies regarding animal leptospirosis, in order to contribute to a better understanding of the epidemiology of animal leptospirosis in Rio de Janeiro, Brazil, as a possible model for other tropical regions.

## Methods

Two internet databases were consulted with the keywords “Leptospirosis”, “*Leptospira*” for the MEDLINE (Medical Literature Analysis and Retrieval System Online), PubMed and SciELO databases. In addition, the Google search engine was used to identify studies outside of the peer–reviewed journal literature. Relevant papers from each bibliographic database and the Google search engine were systematically searched using the following search terms, or derivatives of these, depending on the subject heading terms used by the databases: livestock OR cattle OR horse OR sheep OR goats OR dogs OR “*Rattus norvegicus*” OR pig OR “wild mammals” AND “Rio de Janeiro”. The filters applied to the whole research were Portuguese and English language, and the search interval was January 1990 to January 2013. For all obtained results, full texts were obtained and analyzed. After analysis of the full text of the obtained manuscripts, articles that did not meet the original criteria (animal leptospirosis in Rio de Janeiro, Brazil) of the review were removed.

A total of 15 articles met the inclusion criteria and were studied. They are related to the leptospirosis in rats (*Rattus norvegicus*) captured from an urban area [17] and dogs with clinical suspect of the disease [8,18]. Regarding livestock, there were studies about cows [9,19,20], horses [21-23], goats [4,24], sheep [4] and pigs [25]. Wild mammals kept in the local zoo were also studied [26], as well as golden-lion tamarins from a research center [27] and wild felines [28]. The main limitation of that meta-analysis was that few groups are dedicated to the study of animal leptospirosis in Rio de Janeiro; therefore, the majority of cited articles were conducted by the same research group, reducing the possibility of comparing results and making a broader discussion.

## Background

For the last 20 years, our research group has been dedicated to generating new knowledge regarding the seroepidemiology of animal leptospirosis in Rio de Janeiro, Brazil. Among other species and approaches, since the 1990’s it has been studied rats (*Rattus norvegicus*) captured from an urban area [17] and dogs with clinical indications of the disease [8,18]. In relation to livestock, it was studied cows [9,19,20], horses [21-23], goats [4,24], sheep [4] and pigs [25]. Wild mammals kept in the local zoo were also studied [26], as well as golden-lion tamarins from a research center [27] and wild felines [28].

### Sampling

With the exception of the studies on urban rats and the wild animals, all of the studies were prospective and conducted in the last 20 years. For those, sampling was calculated based on the official population of each species on Rio de Janeiro state and statistical formulae $(\mathrm{ss}=\frac{z^{2}*\left(p\right)*\left(p-1\right)}{C^{2}}$, confidence level of 95%) determining the sampling as well as their distribution was applied, to represent the population of each species in various regions of the state. In the particular case of wild animals and urban rats, the totality of animals which fit the inclusion criteria for each study was tested. Therefore, a total of 47 urban rats, 120 dogs, 875 cows, 695 horses, 1,343 goats, 308 sheep and 351 pigs from all the regions of the state were tested, in addition to 77 mammals (except felines) and 30 wild felines from Rio de Janeiro Zoo and 73 golden-lion tamarins from the Primatology Center of Rio de Janeiro. All studies were previously approved by the Ethics Committee of Universidade Federal Fluminense. Blood samples were collected from the vena jugularis (for rats, direct cardiac puncture was used) into vacuum tubes and allowed to clot at room temperature. At the laboratory, sera was separated by centrifugation and stored at -20°C to be tested as a batch.

### Serology

The microscopic agglutination test (MAT) with live antigens was employed, as recommended [29] (Table 1). Briefly, serum samples were screened at a 1:100 dilution and a collection including 24 serovars from all serogroups was employed as antigens, in order to determine the adequate antigen battery for each animal in that particular region.

**Table 1:** **Twenty-four *Leptospira *** **strains used in microscopic agglutination test (MAT) for serological diagnosis of various animal species in Rio de Janeiro, Brazil**

**Species**	**Serovar**	**Serogroup**	**Reference strain**
*L. borgpetersenii*	Ballum	Ballum	Mus 127
*L. borgpetersenii*	Castellonis	Ballum	Castellon 3
*L. borgpetersenii*	Javanica	Javanica	Veldrat Batavia 46
*L. borgpetersenii*	Mini	Mini	Sari
*L. borgpetersenii*	Tarassovi	Tarassovi	Perepelicin
*L. borgpetersenii*	Whitcombi	Celledoni	Whitcomb
*L. interrogans*	Australis	Australis	Ballico
*L. interrogans*	Autumnalis	Autumnalis	Akiyami A
*L. interrogans*	Bataviae	Bataviae	Van Tienen
*L. interrogans*	Bratislava	Australis	Jez Bratislava
*L. interrogans*	Canicola	Canicola	Hond Utrecht IV
*L. interrogans*	Copenhageni	Icterohaemorrhagiae	M 20
*L. interrogans*	Hardjo	Sejroe	Hardjoprajitno
*L. interrogans*	Hebdomadis	Hebdomadis	Hebdomadis
*L. interrogans*	Icterohaemorrhagiae	Icterohaemorrhagiae	RGA
*L. interrogans*	Pomona	Pomona	Pomona
*L. interrogans*	Pyrogenes	Pyrogenes	Salinem
*L. interrogans*	Wolffi	Sejroe	3705
*L. kirschneri*	Butembo	Autumnalis	Butembo
*L. kirschneri*	Cynopteri	Cynopteri	3522 C
*L. kirschneri*	Grippotyphosa	Grippotyphosa	Moskva V
*L. noguchii*	Panama	Panama	CZ 214 K
*L. santarosai*	Guaricurus	Sejroe	Bov. G
*L. santarosai*	Shermani	Shermani	1342 K

All strains were grown in liquid medium (EMJH for 7–10 days at 28-30°C), free of contamination or auto-agglutination. All samples with agglutinating activity at a 1:100 dilution were considered positive and subsequently titrated against reacting antigens, using serial two-fold dilutions of serum. The endpoint was the highest tube in which 50% agglutination was recorded, leaving 50% free cells compared to a control culture diluted 1/2 in phosphate buffered saline. The highest titre reached was used to suggest the infective serogroup. Since the reliability of MAT in discriminating among serovars of the same serogroup has been reported [30], in the present study we refer to the serological results by serogroup.

## Results

In all species studied, seroreactivity to leptospirosis was very frequent (at varying levels). Therefore, we inferred that the infection is endemic and widely distributed in Rio de Janeiro, Brazil. The seroprevalence rates and the predominant serogroups for each species are shown (Table 2).

**Table 2:** **Anti-*Leptospira *** **sp. antibodies prevalence rates and predominant serogroups for various animal species studied in Rio de Janeiro, Brazil**

**Specie**	**N**	**Seroprevalence (%)**	**Predominant serogroup(s)**	***Reference***
Rats (*R. norvegicus*)	47	17 (36.2)	Icterohaemorrhagiae	[17]
Dogs	120	88 (73.3)	Icterohaemorrhagiae	[18]
Cattle	875	335 (38.3)*	Sejroe	[9,19,20]
Horses	695	275 (39.6)*	Icterohaemorrhagiae/Australis	[21,23]
Goats	1,343	200 (14.9)*	Sejroe	[4,24]
Sheep	308	146 (47.4)	Sejroe	[4]
Pigs	351	232 (66.1)	Icterohaemorrhagiae	[25]
Wild mammals (except felines)	77	29 (37.7)	Icterohaemorrhagiae	[26]
Wild felines	30	4 (13.3)	Icterohaemorrhagiae/Pomona	[28]
Golden-lion tamarins	73	11 (15.1)	Icterohaemorrhagiae	[27]

*Average of the reported prevalence.

The highest prevalence of seroreactivity among all studied species was for dogs (73.3%), followed by pigs (66.1%), both of them predominantly reactive to the serogroup Icterohaemorrhagiae. Horses had 27 and 42.9% (average 39.6%) seroreactivity, mainly to serogroups Australis and Icterohaemorrhagiae.

In ruminants, seroreactivity was highest in sheep (47.4%), followed by cows (23% 36.9 and 46.9%; average 38.3%) and goats (25.9 and 11.1%; average 14.9%). Most seropositive ruminants had reactions against the serogroup Sejroe, which contains the serovar Hardjo, usually reported as the most frequent leptospirosis agent for ruminants worldwide. Wild animals also had a high level of seroreactivity. In regards to the wild mammals from the zoo, antibodies were particularly more common in Canidae, Myrmecophagidae and Procyonidae, with 7 of 9, 5 of 9, and 5 of 9 being positive, respectively. The average of seroreactivity of wild mammals other than felines in the zoo was 37.7%, whereas for wild felines (genus *Herpailurus, Leopardus, Panthera* and *Puma*) it was 13.3%, with the majority of the reactions directed against the serogroups Pomona and Icterohaemorrhagiae. Despite the lower level of seroreactivity in golden-lion tamarins (15.0%), serogroup Icterohaemorrhagiae was the most common agent in these animals. Finally, in urban rats there was 36.2% of seroreactivity, predominantly against the serogroup Icterohaemorrhagiae.

## Discussion

Although serology has some limitations, not only on sensitivity but also specificity and cross reactions, it is still the most widely used tool for large-scale epidemiological studies [6]. Despite inherent limitations of serological testing, we inferred that leptospirosis is endemic and widespread in Rio de Janeiro, since all tested animal species had high seroreactivity. It was not an unexpected result, since studies from other regions of Brazil [13,31-33] as well as from other tropical areas reported leptospirosis as a widespread infection [1,6,11]. Despite this, animal leptospirosis is often neglected and many practitioners, even from endemic regions, are only familiar with the acute syndromes of the disease. Therefore, mainly in livestock, the silent reproductive syndrome is often not investigated, leading to failure to identify the disease. Furthermore, it is noteworthy that tropical regions offer excellent conditions for the survival and spread of leptospires, due to the climate and particularly the rainfall. Those conditions are often associated with poor management practices (rural areas) or poor sanitary conditions (urban cycle) and have been clearly demonstrated to be strongly associated with occurrence of the infection [9,10,12].

After the serogroup distribution was determined for each species, it was apparent that a reduced number of antigens would have detected more than 98% of the seroreactive samples from animals. Therefore, a simplified antigen battery including representants of the more frequent serogroups that occur in Rio de Janeiro for each species was recommended and employed at the laboratory, except for wild animals (Table 3). Since it is well known that human leptospirosis is usually a reflection of serovars that are maintained by the animal population of a region [2,10], it is highly probable that also human leptospirosis can be reliably detected using that simplified antigen battery.

**Table 3:** Recommended antigen battery (serogroups) for microscopic agglutination test for various animal species in Rio de Janeiro, Brazil

**Ruminants**	**Horses**	**Dogs**	**Pigs**
Australis	Australis	Canicola	Australis
Grippotyphosa	Canicola	Icterohaemorrhagiae	Icterohaemorrhagiae
Pomona	Icterohaemorrhagiae	Grippotyphosa	Pomona
Sejroe	Pomona	Pomona	Sejroe
-	Sejroe	-	Tarassovi

Cut-off values varied between epidemiological studies (sampling animals randomly) and diagnostic investigations (testing animals or herds with clinical/reproductive symptoms). Overall, we concluded that, when paired serology is not available (which is particularly common for animal leptospirosis), a single sample reaction with titres ≥400 may be considered as acute leptospirosis [30]. Chronic and host-adapted infection can be accompanied by low titers, whereas acute infections are usually associated with high titers. In addition, the extent to which an infection is considered endemic in a specific region may also be considered for determining an appropriate cut-off point [34]. In that regard in tropical and endemic areas, many groups consider only reactions ≥800 for the diagnosis of acute leptospirosis using one single sample [35]. Our group has investigated the association of titres and clinical signs, together with complementary exams (hematology and biochemistry) and reproductive signs (in livestock). Many laboratories consider 100 as the recommended cut-off titre [29], and more recently, a cut-off of 160 has been suggested for MAT [30]. Nevertheless, that last cut-off (160) was related to laboratory case definition in humans, which are incidental hosts. Therefore, this suggestion cannot be simply extrapolated to animals, due to the chronic cases and to host-adapted infections. Additionally, it is known that infection by host-adapted serovars usually leads to low titres [1,6,32]. Therefore, considering that the disease is endemic in tropical areas, we suggest that, for animal leptospirosis in the tropics, a cut-off of 200 should be considered, although broader studies are required to increase the specificity and consequently the reliability of the diagnosis.

Besides observing that leptospirosis is widely distributed among all the studied species, it is possible to discriminate some epidemiological patterns that can be useful for the understanding of the agent-host-environment interactions under tropical conditions. Basically, there are two major conditions, with two cycles each: the urban/rural condition and the host-adapted/incidental cycle, which may or not be coincident.

The urban cycle is clearly dominated by members of the serogroup Icterohaemorrhagiae, which causes acute clinical disease. Members of this serogroup are maintained by rodents, mainly the Norwegian rat (*Rattus norvegicus*) [36]. They are extremely virulent [37] and cause severe acute illness in several species, including humans [7,13]. Rats captured in Rio de Janeiro were highly infected [17], consistent with findings from other regions [38,39]. In addition to environmental conditions, this certainly contributes to the predominance of this serogroup in all the studied species in the urban cycle. The impact of this serogroup is so important in urban regions that, even with animal species that have their own host-adapted strains, as urban dogs (reservoirs of Canicola), seroreactivity against Icterohaemorrhagiae is much more common than that directed against Canicola, as should be expected. This outcome was not surprising, since urban dogs are constantly exposed to Icterohaemorrhagiae [5,10]. In this scenario, dogs are susceptible to the same conditions as humans (flood risk areas and waste accumulation sites), which contribute to the presence of the agent in the environment, and therefore increase the zoonotic risk [8,15,40]. Serogroup distribution among wild mammals at the zoo was of particular interest. With the exception of wild felines, which are considered resistant to Icterohaemorrhagiae strains [28], several species, from different origins and with a great diversity of habits, the predominant seroreactivity was once more against Icterohaemorrhagiae [26]. Therefore, we hypothesize that, in the urban cycle of leptospirosis, environmental conditions that favor infection by Icterohaemorrhagiae are very important and have a greater impact on the epidemiology of the disease than differences among host species.

This scenario was not only applicable to Rio de Janeiro; it was recently reported that in the urban area of Salvador, Brazil [41], as well as in other tropical islands [38], where marmosets are predominantly seroreactive against that serogroup. Therefore, similar associations are expected in many tropical regions of the world. Indeed, dogs from a town in Amazon rain forest [42], as well as captive animals in Peruvian Amazon [43] were seroreactive against Icterohaemorrhagiae, and not to serogroups maintained by wild animals, as might be expected.

In relation to the rural cycle of the infection, differences between infections caused by incidental or adapted serogroups are clearer. Among livestock, the most studied species are ruminants (cattle, sheep and goats). In those species, serogroup Sejroe was highly predominant, consistent with studies conducted in other tropical [1,6,44] and even non-tropical areas [45,46], as well as in other regions of Brazil [31,47,48]. Members of this serogroup, mainly Hardjo, are known to be adapted to ruminants [16] and its maintenance within a herd may also occur by animal-animal direct transmission. Although infection by Hardjo in cattle has been reported as less dependent on environmental conditions [44], it may not be absolutely valid for the tropics [19]. Therefore, we have proposed that under tropical conditions, a successful leptospirosis control program, in addition to vaccinations and antimicrobial treatment, should also include an investigation of environmental and herd management practices in order to identify factors likely to affect transmission and prevalence of the disease [9,19].

Incidental infection in ruminants are infrequent and usually leads to outbreaks and severe clinical illness, whereas infections by adapted strains are often chronic, with only mild symptoms, particularly with regards to reproductive function [6,49]. Pomona, the most commonly reported serogroup for incidental infections in cattle from 1970–1980, is currently only rarely detected [44]. We infer that this is due to improved husbandry practices, mainly the reduction of the habit of co-grazing cattle with other species, particularly pigs. In that regard, pigs act as reservoirs of that serogroup and their presence on cattle farms has been strongly associated with the occurrence of incidental leptospirosis [9].

Therefore, in contrast to in the urban cycle, where incidental acute infection by Icterohaemorrhagiae is predominant, in the rural cycle, chronic and mild infections determined by adapted strains, mainly from serogroup Sejroe, seem to be more common [20]. This fact may explain the misdiagnosis of the infection among practitioners that are not familiarized with these details, with significant impacts on animal production as well on public health. The study of animal leptospirosis is directly related to the understanding and prevention of the infection in humans, mainly in tropics [38]. Nevertheless, in the urban scenario, human leptospirosis is due to an interaction among the presence of reservoir (mainly urban rodents) of leptospires (e.g. Icterohaemorrhagiae serogroup members), a highly contaminated environment, with high rates of exposure to the agent (e.g. household clustering), as observed in slum communities. In contrast, in rural areas, serovars that most commonly infect livestock (e.g. Hardjo), only rarely cause human infections [10,13,15]. Furthermore, the role of rodents in the rural cycle has been neglected and more studies are needed.

## Conclusion

In conclusion, this paper discusses the current knowledge on animal leptospirosis in Rio de Janeiro and demonstrates important differences between urban and rural cycles of the infection, depicting the serogroup distribution in both cycles and in various species. Identification of the prevailing serogroups and of their animal reservoirs is essential for understanding agent-host-environment interactions under tropical conditions.

## Competing interests

The authors declare that they have no competing interests.

## Authors’ contributions

GM participated in the design, conducted the analysis and drafted the manuscript. WL carried out the coordination and critical and final review of the manuscript. Both authors read and approved the final manuscript.
